# Disturbances in Body Ownership in Schizophrenia: Evidence from the Rubber Hand Illusion and Case Study of a Spontaneous Out-of-Body Experience

**DOI:** 10.1371/journal.pone.0027089

**Published:** 2011-10-31

**Authors:** Katharine N. Thakkar, Heathman S. Nichols, Lindsey G. McIntosh, Sohee Park

**Affiliations:** 1 Department of Psychology, Vanderbilt University, Nashville, Tennessee, United States of America; 2 Department of Psychiatry, Vanderbilt University Medical College, Nashville, Tennessee, United States of America; Ecole Polytechnique Federale de Lausanne, Switzerland

## Abstract

**Background:**

A weakened sense of self may contribute to psychotic experiences. Body ownership, one component of self-awareness, can be studied with the rubber hand illusion (RHI). Watching a rubber hand being stroked while one's unseen hand is stroked synchronously can lead to a sense of ownership over the rubber hand, a shift in perceived position of the real hand, and a limb-specific drop in stimulated hand temperature. We aimed to assess the RHI in schizophrenia using quantifiable measures: proprioceptive drift and stimulation-dependent changes in hand temperature.

**Methods:**

The RHI was elicited in 24 schizophrenia patients and 21 matched controls by placing their unseen hand adjacent to a visible rubber hand and brushing real and rubber hands synchronously or asynchronously. Perceived finger location was measured before and after stimulation. Hand temperature was taken before and during stimulation. Subjective strength of the illusion was assessed by a questionnaire.

**Results:**

Across groups, the RHI was stronger during synchronous stimulation, indicated by self-report and proprioceptive drift. Patients reported a stronger RHI than controls. Self-reported strength of RHI was associated with schizotypy in controls Proprioceptive drift was larger in patients, but only following synchronous stimulation. Further, we observed stimulation-dependent changes in skin temperature. During right hand stimulation, temperature dropped in the stimulated hand and rose in the unstimulated hand. Interestingly, induction of RHI led to an out-of-body experience in one patient, linking body disownership and psychotic experiences.

**Conclusions:**

The RHI is quantitatively and qualitatively stronger in schizophrenia. These findings suggest that patients have a more flexible body representation and weakened sense of self, and potentially indicate abnormalities in temporo-parietal networks implicated in body ownership. Further, results suggest that these body ownership disturbances might be at the heart of a subset of the pathognomonic delusions of passivity.

## Introduction

*“I felt like an alien that was being used to manipulate humans by other aliens. It felt like someone was looking through my eyes without my consent.” –Study participant*

Early descriptions of schizophrenia stress disturbances in self-processing [1], and some posit that altered sense of self is central to both positive and negative symptomatology [2]. Anomalous self-awareness is evident in passivity phenomena, in which the patient does not experience himself as the agent of his actions, instead attributing them to an external source. Disturbances in self-processing in schizophrenia have been discussed in the psychoanalytic tradition [3], but do not easily lend themselves to empirical study.

Self-awareness can be separated into body ownership and agency. Body ownership refers to “the perceptual status of one's own body, which makes bodily sensations seem unique to oneself” [4], and it contributes to a sense of self and a developmental basis for a psychological identity [5]. Agency refers to the subjective experience of being the initiator of one's actions. Impairments in a sense of agency are thought to underlie several psychotic symptoms. Yet, based on patient report, body ownership is presumed to be largely intact. However, experimental support for intact body ownership in schizophrenia is limited, and clinical observations do not preclude subtle disturbances in body ownership that may contribute to an anomalous sense of agency.

A recent surge of interest in the cognitive neuroscience of self-awareness has cultivated empirical investigations of body ownership, for example with the rubber hand illusion (RHI). Watching a rubber hand being stroked while one's own unseen hand is stroked simultaneously often leads to a sense of ownership over the rubber hand and a shift in perceived position of the real hand toward the rubber hand [6]. The RHI is reduced or absent when tactile stimulation of the real and rubber hands is asynchronous or when the rubber hand is spatially incongruent with the real hand [7]. Thus, visuo-tactile correlation is necessary, but not sufficient, to induce the RHI; it is also dependent on the representation of one's own body.

The RHI is typically measured with self-report questionnaires, and the shift in perceived hand location before and after stimulation (“proprioceptive drift”). A physiological correlate of the RHI involves a limb-specific drop in hand temperature [8], with cooling being observed in the stimulated, but not unstimulated, hand. Cooling in the stimulated hand was related to self-reported RHI strength, and importantly, hand temperature was unchanged during asynchronous tactile stimulation.

A recent study [9] showed that the RHI is intensified and has a more rapid onset in schizophrenia. However, these authors only administered synchronous stimulation and did not measure proprioceptive drift, so it is unclear whether these findings reflect a more liberal threshold to report illusion onset and/or differences in response biases on the questionnaire in patients.

The major aim of the present study was to systematically investigate disturbed body ownership with the RHI in schizophrenia and its relationship to delusions and hallucinations. The strength of the RHI was measured using self-reported intensity of perceptual experiences, proprioceptive drift, and changes in hand temperature. In healthy controls, we also examined RHI strength in relation to psychometric schizotypy. Schizotypy refers to the personality traits that are related to symptoms of schizophrenia and imply a latent liability for the disorder [10]; it was assessed using a self-report measure. We hypothesized that a stronger RHI in schizophrenia would be consistent with the notion of a weaker or more flexible sense of body ownership.

## Methods

### Ethics Statement

This study was conducted according to the principles expressed in the Declaration of Helsinki. The Vanderbilt Institutional Review Board approved the study protocol and informed consent procedure. After complete description of the study to the subjects, written informed consent was obtained.

### Participants

Twenty-four schizophrenia outpatients (SZ) were recruited from a psychiatric facility in Nashville, TN. Diagnoses were made according to Diagnostic and Statistical Manual of Mental Disorders, Fourth Edition (DSM-IV) criteria using structured clinical interviews (SCID-IV). All patients were medicated. Symptoms were assessed with the Brief Psychiatric Rating Scale (BPRS) [11], the Scale for the Assessment of Positive Symptoms (SAPS) [12], and the Scale for the Assessment of Negative Symptoms (SANS) [13]. Twenty-one healthy control participants (HC) without a history of DSM-IV Axis I disorder or use of psychotropic medications were recruited from the same community by advertisements. The Schizotypal Personality Questionnaire (SPQ) [14] was administered to HC. All subjects were screened for neurological disorders, drug use and past head injury.

Intelligence was assessed with the Adult North American Reading Test (ANART) [15]. Handedness was assessed using the Modified Edinburgh Handedness Inventory [16]. All subjects had normal or corrected-to-normal vision. The two groups were matched for age, sex, and handedness. Although HC had a higher estimated IQ and more education, both groups were well within the normal IQ range and, on average, SZ had completed one year of college. Demographic characteristics and SPQ scores are outlined in Table 1. All subjects were paid for their participation.

**Table 1:** Demographic characteristics of the patient and control groups.

	Patients (n = 24) Mean (s.d.)	Controls (n = 21) Mean (s.d.)	t	p
Age	41.7 (8.3)	40.1 (9.1)	0.6	0.53
Sex	9F/15 M	10 F/11 M	Phi = 0.5^1^	0.49
Handedness^2^	60.0 (57.5)	82.6 (43.0)	1.5	0.15
Estimated IQ (ANART)	101.1 (9.9)	106.5 (6.3)	2.1	0.04
Years of education	13.0 (2.3)	15.8 (2.3)	3.8	0.0004
SPQ-Positive Syndrome	N/A	3.0 (3.2)		
SPQ-Negative Syndrome	N/A	5.2 (4.2)		
SAPS	14.3 (9.6)	N/A		
SANS	25.0 (17.2)	N/A		
BPRS	14.1 (8.1)	N/A		
Chlorpromazine (CPZ) equivalent dose^3^	362.8 (318.9)	N/A		
Psychotropic Medications	4 typical antipsychotic drugs	N/A		
	19 atypical antipsychotic drugs			
	1 antidepressant			
Duration of illness (years)	20.4 (10.1)	N/A		

1 The Phi value is the result of a Fishers exact test.

2 Edinburgh handedness inventory : −100 completely sinistral to +100 completely dextral.

3 Chlorpromazine (CPZ) equivalent dose of antipsychotic medication (mg/kg/day).

N/A  =  not applicable.

### Materials

**Figure 1:**
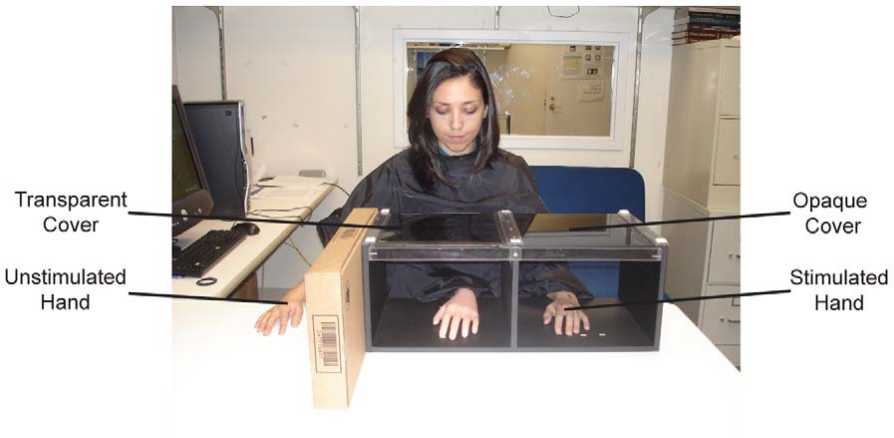
Experimental set-up.

Subjects were seated in front of a two-compartment, open-ended box (Figure 1). One compartment had a transparent cover, and the other had an opaque cover. Subjects placed one hand in the opaque compartment and the other hand behind a barrier on the table beside the box, rendering both hands hidden from the subject's view. A lifelike rubber hand was positioned in the transparent compartment. Although visual similarity between the rubber hand and participant's hand does not have an effect on the RHI [17], a neutral density filter was placed over the cover to de-emphasize the color of the rubber hand. A cape was placed around the subject to cover his or her arms and the end of the rubber hand.

Skin temperature measurement was modeled after Moseley, et al. [8] and obtained using a handheld non-contact thermometer (Fluke Corporation, Everett, WA) from three points on the each hand (below the second and fifth digits and on the wrist), marked with washable marker.

### Procedure

Prior to tactile stimulation, four measurements of perceived index finger location of the hidden hand under the opaque cover were taken. A ruler was placed on top of the box, and subjects were asked to verbally indicate their perceived index finger position. Different rulers, each offset by a random length, were used to prevent subjects from reciting the same number for each measurement. Baseline skin temperature was recorded at the three locations on the stimulated and unstimulated hand. Following pre-stimulation measurements, the experimenter brushed the index finger of the rubber hand and that of the subject's invisible hand with a paintbrush at approximately one stroke per second. The subject was instructed to watch the rubber hand and report any change in sensation they might experience. The fingers were brushed for 3 minutes, either synchronously or asynchronously (180 degrees out-of-phase). During stimulation, a second experimenter recorded skin temperature at the three locations of each hand at 1, 2, and 3 minutes. Following stimulation, four measurements were taken of the subject's perceived index finger location of the hidden hand. See *Figure 1* for experimental set-up. In addition, a movie of the procedure is available at http://vanderbilt.edu/parklab/Projects.html.

**Figure 2:**
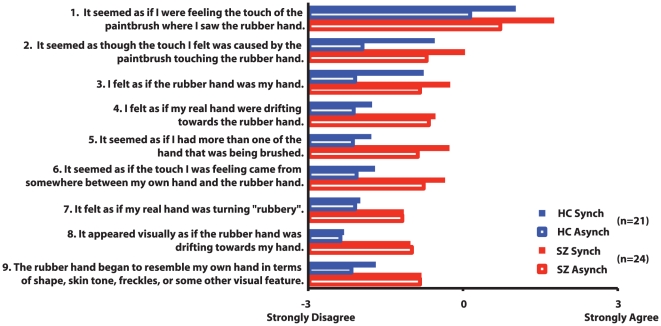
Mean rating for each item in the self-report questionnaire for each group following synchronous and asynchronous tactile stimulation.

Finally, the subject was asked to provide an open-ended description of the experience and was given a standard questionnaire [6] to rate the occurrence of nine perceptual effects (*Figure 2*) on a 7-point Likert scale from -3 to 3. This procedure was performed four times, for both synchrony conditions in both hands. Trial order was counterbalanced across subjects.

### Analysis

#### Proprioceptive Drift

Proprioceptive drift was quantified as the difference between mean perceived index finger location before and after tactile stimulation. Positive numbers indicate drift toward the rubber hand.

#### Skin Temperature

Temperature was averaged across the three locations on both stimulated and unstimulated hands at each of the three measurement time points. The change in temperature for each hand in each condition was quantified as the temperature difference between baseline and the average of the three stimulation time points. Negative scores indicate cooling. We collapsed across time points because illusion strength varies across the stimulation period and the strength of the illusion was found to correspond with temperature modulation [8]. Since it was not feasible to track RHI strength at each time point, we averaged across them. Temperature data from one patient was lost due to recording error.

#### Statistical Analyses

Separate repeated-measures ANOVAs were conducted on self-reported illusion strength from the questionnaire, proprioceptive drift, and temperature modulation, with diagnosis entered as a between-group variable and stimulated hand (right or left) and synchrony condition (synchronous or asynchronous) entered as within-subject variables. For questionnaire data analysis, item was also entered as a within-group variable. For temperature modulation, stimulation condition (stimulated or unstimulated) was also entered as a within-subjects variable. Spearman rank-correlation coefficients were used to evaluate the association between RHI measures and the severity of symptoms in SZ, and SPQ subscale scores in HC.

## Results

### Self-report RHI Questionnaire

There was a main effect of synchrony (F(1,43) = 16.5, p = 0.0002). Paired t-tests indicated that, in both groups, the strength of the RHI was greater during synchronous than asynchronous stimulation (t(44) = 4.0, p = 0.0002). Although there was a significant effect of group (F(1,43) = 5.7, p = 0.02), there was no group-by-synchrony interaction effect (F(1,43) = 0.7, p = 0.4). SZ endorsed RHI-related perceptual effects more strongly than HC in both synchronous (t(43) = 2.0, p = 0.05) and asynchronous (t(43) = 2.7, p = 0.01) conditions. There were significant item (F(8,344) = 22.4, p<0.0001) and synchrony condition-by-item effects (F(8,344) = 5.6, p<0.0001). After Bonferroni correction for multiple comparisons, only items 1–3 and 6 were rated more strongly in the synchronous condition (*Figure 2*). Please note that these first three items have been found to be most reliably endorsed following synchronous stimulation and our results replicate those of others [6].

### Proprioceptive drift

**Figure 3:**
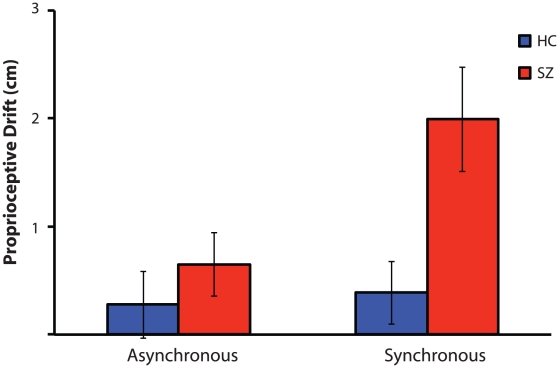
Mean proprioceptive drift for each group following synchronous and asynchronous tactile stimulation. Vertical bars denote standard error of the mean.

Results are displayed in *Figure 3*. There were significant main effects of group (F(1,43) = 5.1, p = 0.03) and synchrony condition (F(1,43) = 7.5, p = 0.009), with proprioceptive drift being greater in SZ and larger following synchronous than asynchronous stimulation. Importantly, there was also a significant group-by-synchrony interaction (F(1,43) = 5.4, p = 0.02). Greater proprioceptive drift was observed in SZ following synchronous (t(43) = 2.8, p = 0.009), but not asynchronous stimulation (t(43) = 0.9, p = 0.39). Paired t-tests indicated that although proprioceptive drift was greater following synchronous than asynchronous stimulation in SZ (t(23) = 3.3, p = 0.003), this effect was not observed in HC (t(20) = 0.3, p = 0.74). These differences in proprioceptive drift cannot be explained by differences in perceived baseline location, as the initial error did not differ between groups (t(43) = 0.4, p = 0.71) or synchrony conditions (t(44) = 1.1, p = 0.28).

### Temperature Modulation

**Figure 4:**
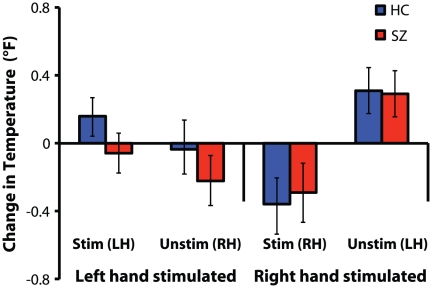
Mean change in skin temperature in both the unstimulated and stimulated hand during right and left hand stimulation for patients and controls. Vertical bars denote standard error of the mean.

Results are displayed in *Figure 4*. A significant effect of stimulation on temperature change was observed (F(1, 42) = 4.7, p = 0.04), such that temperature dropped in the stimulated hand and rose in the unstimulated hand. However, this effect was moderated by hand of stimulation, indicated by a stimulation-by-hand interaction (F(1,42) = 21.8, p<0.0001). Significant heating of the unstimulated hand (t(43) = 2.3, p = 0.02) and cooling of the stimulated hand (t(43) = 2.8, p = 0.008) were only observed when the right hand was brushed. However, an analysis of baseline temperature revealed a significant effect of both hand (F(1,42) = 41.1, p<0.0001) and stimulation condition (F(1,42) = 30.1, p<0.0001). Temperature was higher in the left hand, and baseline temperature in the stimulated hand was greater than in the unstimulated hand. However, see *Data S1* for results from a control experiment that indicated the limb-specific temperature modulations observed in the main experiment were not caused by idiosyncratic parameters of the experimental environment, but instead, were related to RHI induction.

### Clinical symptoms and schizotypy

We examined correlations between clinical symptoms and the following measures in patients: self-reported illusion vividness for questions 1–3 for both synchrony conditions (since these item scores were significantly different between synchrony conditions in the current and previous studies), mean proprioceptive drift in the two synchrony conditions, and mean change in temperature in the stimulated and unstimulated hand during right hand stimulation (since no stimulation effect on temperature was observed when the left hand was stimulated). In HC, we examined the relationship between these measures and schizotypy. In patients, we explored the relationship between these measures and BPRS, SAPS, and SANS. Because we were specifically interested in the relationship between body ownership and hallucinations and delusions, we also investigated correlations between RHI measures and individual items from the SAPS subscales. SAPS subscale and individual item scores were missing from one patient.

In HC, self-reported vividness of the illusion during both synchronous and asynchronous stimulation was associated with increased positive (synchronous: r_s_ = 0.61, p = 0.003; asynchronous: r_s_ = 0.49, p = 0.03) and negative (synchronous: r_s_ = 0.59, p = 0.005; asynchronous: r_s_ = 0.62, p = 0.003) schizotypy.

In SZ, self-reported RHI strength in the synchronous, but not asynchronous, condition was related to increased score on the hallucinations subscale of the SAPS (r_s_ = 0.48, p = 0.02). Although there were no significant correlations with the overall delusions subscale score, self-reported illusion strength in the synchronous condition was related to increased delusions of reference (r_s_ = 0.65, p = 0.0008) and delusions of control (r_s_ = 0.53, p = 0.009), and reduced scores for somatic delusions (r_s_ = −0.44, p = 0.03). Further, skin temperature increase in the unstimulated hand during right hand stimulation was related to increased delusions of thought insertion (r_s_ = 0.41, p = 0.05). No significant correlations with BPRS and SANS scores were observed.

### Case Report

One individual with schizophrenia reported an out-of-body experience (OBE) during RHI induction with synchronous stimulation applied to the left hand. Because observation of a spontaneous OBE in the laboratory is extremely rare, we decided to conduct a case report in depth. We refer to this participant as R.M. throughout the case report. R.M. agreed to return for an additional visit to investigate whether his OBE was replicable and to obtain a more detailed timeline of his subjective experience.

#### Experimental set-up and procedure

Set-up and procedure were identical to that outlined above. Since R.M. reported an OBE during the synchronous condition for the left hand during the initial experiment, we repeated the experimental procedure on a separate day. In the follow-up procedure, synchronous stimulation was only administered to the left hand, and the stimulation period was extended to 10 minutes.

After the follow-up session, R.M. was asked a series of questions about the phenomenological details regarding previous OBEs and the OBEs he experienced during the rubber hand illusion. These questions were based on a published report of the phenomenological correlates of OBEs in neurological patients [18]. Demographics and medical, psychiatric, and social history were also obtained.

A brief, comprehensive neuropsychological examination was administered. The testing battery included the Repeatable Battery for the Assessment of Neuropsychological Status [19], which includes five subscales: Immediate Memory, Visuospatial/Construction, Language, Attention, and Delayed Memory. Trails A and B and the Stroop (Golden Version) were administered to assess processing speed and executive functions. Demographically-normalized scores were computed.

#### Participant

R.M. is a 55-year-old, right-handed, Caucasian male who was diagnosed with schizoaffective disorder at the age of 23. Since his first hospitalization in 1979, he estimated the number of psychiatric hospitalizations to be around 15–20, with the most recent in May of 2009. R.M. was free of neurological disorder, history of seizures, and history of ECT. He subject reported one head injury with loss of consciousness at the age of 16, but he sustained no internal bleeding and there was no evidence of cognitive sequelae following the injury. R.M. did not meet criteria for any alcohol or substance abuse or dependence and reports smoking marijuana only twice in his lifetime.

R.M. holds a Master's degree and was a reporter until 2002. Since then he has been employed in restaurants, and recently has been volunteering as an archivist and seeking employment as a freelance writer.

Vision and hearing were adequate for testing. Recently, R.M. received eye surgery for glaucoma and indicated normal acuity at the time of the experiment. He also reported Tarsal tunnel syndrome in his left ankle; no additional significant medical history was reported. His current medications included thiothixine (3 mg daily), fenofibrate for cholesterol, omeprazole for stomach acid, and travoprost ophthalmic solution and betaxolol eye drops to manage his glaucoma.

Overall, R.M's neurocognitive functioning was within normal range. He had significant psychomotor slowing, which is consistent with a schizophrenia diagnosis and neuroleptic use. Memory, visuospatial processing, construction, language, and executive functions (inhibition, context switching) were grossly intact. His estimated premorbid IQ was in the superior range at 120.

#### Open-ended description of RHI

In the initial rubber hand experiment, following synchronous stimulation of the right hand, R.M. reported the sense that the rubber hand felt like his hand, and eventually that his real hand moved to the position of the rubber hand at into the trial. He reported the same sensation during synchronous stimulation of the left hand. After a while, he reported that he felt he was “out of his body”, and was hovering above and looking down on himself. Before the end of the 3-minute tactile stimulation period, he reported that he had returned to his body. During both hands of the asynchronous condition, he reported that the feeling of the rubber hand was his own fluctuated and was less strong than during synchronous stimulation. He also reported that he sometimes felt that the unstimulated hand was in the same location as the rubber hand.

During the follow-up session where we synchronously stimulated the left hand for approximately ten minutes, the subject reported a fluctuating sensation of ownership of the rubber hand. He also reported an OBE, which evolved into a perception of himself and the experimenter hovering above the table. Below is his timeline (in minutes and seconds) of his self-reported experience:

0:48 Reports: *“This is my hand; it looks like my hand”*

1:07 Reports feeling of ownership over rubber hand disappearing

1:20 Report that the rubber hand feels and looks like his hand

1:40 Reports fluctuating feeling of ownership over rubber hand

1:46 Reports: *“This is my hand; looks like my hand”*

2:10 Reports that the knuckles of the rubber hand sometimes look like his own

2:23 Sees his own wrinkles in the rubber hand

2:49 Reports: *“Really feels like my hand”*

3:47 Reports imagining things he could do with the hand, like play the violin

4:04 Reports that the rubber hand, *“looks like rubber hand again”*

4:20 Reports that the rubber hand doesn't feel like his hand anymore

5:02 Reports *“strange feeling; that out of body deal”*

5:37 Reports that he and the experimenter are levitating

6:40 Reports that he and the experimenter are *“turning in a circle; rubber hand looks like my hand”*

7:07 Reports, *“feels like we're a foot off the floor, turning in a circle”*

7:39 Reports, *“feels like we're coming back down; felt like there wasn*'*t a floor beneath my feet”*

8: 27 Reports being back on ground

9: 04 Reports that the rubber hand looks like rubber hand, not his hand

#### Description of RHI-induced OBEs

Visual phenomenology: In both his past and RHI-induced OBEs, he described his visuo-spatial perspective in a prone-positioned, elevated second body outside the physical body. He described them as vivid and veridical, and he reported seeing his whole body and other objects in the room in their actual positions. However, he only saw the people and objects in his immediate space (i.e. the experimenter performing the tactile stimulation and the table). In both RHI-induced OBEs, he reported his back to the ceiling, looking at himself seated. Although he reported only seeing from one visuo-spatial perspective during the first OBE and then “snapping out of it” after about 1 minute, he said there was a “duality” about the second OBE episode, where he alternated between his seated and elevated visuo-spatial perspectives and gradually came back to his body.

Non-visual phenomenology**:** In the second RHI-induced OBE, he reported numbness in his feet and hands that preceded the OBE, but this did not occur for the first RHI-induced OBE, nor his past OBEs. For the RHI-induced OBEs, he reported a feeling of elevation but no experience of rotating into the inverted position. Although during the first OBE he felt fearful because he thought he might be going into a psychotic episode (see section on OBE history) and was relieved after he came out of the OBE, he reported the second OBE was pleasant and that he wanted the feeling to come back. Interestingly, he conveyed that for both OBEs, there was “a silence about it”, and he could not hear anything. It is possible that during OBEs, auditory processing is suppressed, although we are not aware of other reports of this phenomenon. He could still feel the tactile sensation of the brush. He reported that although his second body was complete, he did not really think about or acknowledge that body.

#### OBE history

During the extended interview, R.M. reported that has had too many OBEs to count. Typically, they occur before he falls asleep and last approximately 10 minutes. He has had about 12 OBEs while he was awake and alert, and they have always preceded a psychotic episode. Those OBEs last approximately 15 minutes at first, but get longer as his psychotic episode progresses. He reported that his first OBE happened when he was at church camp at age 16. R.M. indicated that his RHI-induced OBEs felt more like the OBEs he has while falling asleep. On the other hand, the OBEs he experiences while awake begin in a similar manner, but then become qualitatively different. He described the feeling of elevation that progresses into religious delusions in which he talks to angels and demons in the sky. He noted that he eventually feels a greater sense of ownership over his ‘second body’ and described this ‘second body’ as a puppeteer and his real body as a marionette. He said these OBEs come and go for about 4-5 days, until he is eventually hospitalized. Thus, R.M. described an *explicit* link between body disownership and psychosis.

After the completion of this experiment and interview, he requested, and was given further information about the OBE, including peer-reviewed journal articles. He then recognized that his experiences may have a tangible cause and interestingly he did not have another psychotic episode even though he had experienced an awake OBE during this experiment. At the time of the final preparation of this article, 10 months after he had participated in this study, he was still in remission, was working part-time, and had a paper accepted for publication in an academic journal. Thus, in this particular case, gaining insight into the etiology of anomalous experiences appears to be therapeutic. However, it must be noted that R.M. is a very high-functioning individual with exceptional verbal skills and insight into his conditions. Furthermore, had we known that there was a link between awake OBEs and psychotic episodes for R.M., we certainly would not have conducted this case study. To be cautious, future studies of RHI in schizophrenia-spectrum should consider screening out those who are prone to OBEs if their OBEs are correlated with psychotic episodes.

## Discussion

The RHI was stronger in individuals with schizophrenia than in healthy participants. On a self-report questionnaire, patients reported this illusion as more vivid than healthy controls, both in synchronous and asynchronous stimulation conditions. Further, self-reported vividness of the illusion was associated with increased positive and negative schizotypy in HC. In patients, vividness of the illusion, during synchronous stimulation only, was associated with elevated hallucinations, consistent with previous findings [9] as well as delusions of reference, and delusions of control, and counter intuitively, reduced report of somatic delusions. Additionally, SZ experienced greater proprioceptive drift during synchronous, but not asynchronous, stimulation. Finally, we observed stimulation-dependent changes in skin temperature. During right hand stimulation, temperature dropped in the stimulated hand and rose in the unstimulated left hand. This effect did not differ across groups or synchrony conditions. Together, these findings are consistent with a weaker or more flexible sense of body ownership in schizophrenia.

These data support the finding of increased RHI vividness in SZ [20]. Across groups, the RHI was subjectively stronger during synchronous stimulation, but we also observed that the RHI was greater in SZ compared with HC during both synchronous and asynchronous stimulation. Greater vividness of the illusion in SZ compared with HC in the asynchronous condition could indicate increased response bias or suggestibility in the patients. However, an alternative explanation is that asynchronous stimulation may be perceived as less temporally asynchronous, or even synchronous, by patients due to a coarser window of temporal integration. There is evidence to indicate that SZ have a longer time window in which they perceive two temporally separate unimodal and bimodal visual and auditory events as simultaneous [21]. Additional evidence that group differences in the RHI are not driven by response biases or suggestibility comes from the novel analysis of proprioceptive drift. Patients and controls showed equal proprioceptive drift in the asynchronous condition, but drift in the synchronous condition was nearly three times as large in SZ. Thus, data from both self-report and proprioceptive drift support a more robust RHI in schizophrenia.

Although self-reported intensity of the illusion was stronger in both synchrony conditions, there was a group difference in proprioceptive drift only for the synchronous condition. Principal components analyses of self-report questions indicate that ownership and location shifts are dissociable components of the RHI [22], a finding which has been supported by recent experimental data [23]. Functional MRI data also suggest that ownership and location shifts are dissociable, and point to a relationship between ventral premotor activation and subjective report [24] and inferior parietal lobule (IPL) activation during recalibration of perceived hand position [24], [25]. Since the self-report questionnaire contains items related to ownership, it is possible that activity in premotor cortex corresponds with feelings of ownership over the rubber hand, and IPL activation is related to proprioceptive drift [24], [25]. This idea is bolstered by the finding that TMS applied over right IPL reduced proprioceptive drift, but not subjective report of RHI intensity [26]. In SZ, body ownership could be even more flexible than perceived location of the body, resulting in a strong subjective sense of the illusion, even when tactile stimulation is asynchronous.

Although we did not observe group differences in skin temperature modulation, our finding of cooling in the stimulated right hand during RHI induction partly replicated the findings of Moseley, et al. [8]. However, contrary to prior findings, no differences in temperature modulation between synchrony conditions were observed. Additionally, temperature in the stimulated hand was warmer than the unstimulated hand at baseline (i.e., before brushing occurs). The cause of this baseline difference is unclear, and might be due to arousal, or orienting effects [27]; data from a control experiment rule out environment-specific factors (See *Data S1*). Data from this control experiment also indicate that the observed cooling of the stimulated right hand and warming of the unstimulated left hand, are not caused by environmental confounds. Moseley and colleagues [8] posit that skin cooling is related to a sense of disownership of the stimulated hand; however, temperature modulation might also result from changes in arousal or orienting. These hypotheses are not necessarily mutually exclusive. Lateralization of skin temperature changes is curious; it might be related to hemispheric contributions to body illusions [18], [28], [29] and should be explored in future neuroimaging or lesion studies.

### Potential Mechanisms

Based on current findings, several non-mutually exclusive hypotheses for mechanisms underlying a stronger RHI in SZ emerge. The RHI may arise from bottom-up multisensory integration of conflicting signals, with visual information overwriting the relatively weaker proprioceptive input. However, the RHI is significantly modulated in a top-down fashion by the internal model of the body, such that the illusion is typically not observed when the rubber hand is spatially incongruent with the real hand [7] or when non-corporeal objects are used [7], [30]. One possibility is that disturbances in multisensory integration lead to an exaggerated RHI in SZ, such that visual information is overweighed because of altered proprioception [31]. However, this hypothesis is inconsistent with findings of reduced influence of visual information on proprioceptive [32] and auditory [32], [33] signals in schizophrenia. Alternatively, SZ might have a weaker or more flexible internal model of their body, making them more susceptible to the illusion. This would be consistent with the anecdotal observations in this and a previous study [20], that some patients experience the illusion even before tactile stimulation begins.

Regarding neural mechanisms, fMRI [25] and TMS [34] data implicate the right temporoparietal junction (TPJ) and IPL in body ownership. Further, somatoparaphenia, a neurological syndrome associated with dis-ownership of a body part, is most often associated with lesions in these regions [29], and right TPJ seizure focus is associated with OBEs [18], [28]. Although there is relatively little data on IPL and TPJ function and structure in schizophrenia, behavioral and neuroimaging studies implicate possible IPL and TPJ disturbance in schizophrenia spectrum disorders [35], [36].

A stronger RHI in schizophrenia might also be related to NMDA glutamate hypofunction. The glutamate hypothesis of schizophrenia is promising, due to the observation that NMDA antagonists mimic both positive and negative symptoms and can account for the developmental profile of schizophrenia [37]. Interestingly, a recent study found that ketamine administration to healthy volunteers led to an increase in self-reported RHI vividness and proprioceptive drift in both synchronous and asynchronous stimulation conditions [38], and frequency of ketamine use has been found to correlate more strongly with OBE frequency than other drugs [39].

### Limitations

One limitation of this study is that we cannot eliminate potential effects of antipsychotic medication, but it seems unlikely that neuroleptic effects drive these findings. Ketamine administration results in a stronger RHI in healthy volunteers [38]. Pretreatment with an atypical antipsychotic blocks ketamine-induced metabolic activation in the rodent brain [40] and attenuates ketamine-induced increases in positive symptoms in schizophrenia [41]. Furthermore, CPZ equivalent dose was not related to any of the RHI measures in our patients (all p's >0.10). Finally, self-reported RHI strength correlated with schizotypy in unmedicated healthy participants, a finding that cannot be attributed to medication.

Another limitation of the current study is that the illusion was not very robust in HC. Although we did observe drift towards the rubber hand in healthy controls, this effect was small during both synchronous (Cohen's d = 0.29) and asynchronous (Cohen's d = 0.19) brushing. One probable reason for this finding is the relatively short period of tactile stimulation compared to prior studies [6]. We chose the briefest possible duration of stimulation from past reports of RHI in order to minimize the total testing time for our participants. Nevertheless, even with the brief stimulation duration, psychometric schizotypy was associated with strength of the RHI in healthy participants. It is also evident from *Figure 2* that the mean self-reported RHI intensity for many questions fell below zero in both groups. However, it is important to note that a rating of 0 does not mean that the particular perceptual effect was absent. Rather, a score of −3 (definitely disagree) indicates the absence of the effect.

With regard to temperature data, our task parameters might not be optimal for measuring subtle temperature modulations. Moseley, et al. [8] used a longer stimulation period and began measuring skin temperature only *after* subjects reported the onset of the illusion. Further, they measured temperature at shorter time intervals. Future studies investigating RHI-induced physiological changes in schizophrenia should take these methodological considerations into account.

### Implications

Correlations between schizotypy and RHI strength suggest a weaker sense of body ownership in those who may share latent liability for schizophrenia, although we cannot rule out response bias effects. In SZ, correlations between RHI strength and positive, but not negative symptoms, suggest that disturbed body ownership might contribute to psychotic symptoms. Indeed, one patient reported an explicit link between body disownership and psychotic episode onset. Since our sample included fairly asymptomatic patients, future studies should examine the RHI in patients with more severe symptoms. Nevertheless, these results present an intriguing potential link between body ownership disturbances and the agency abnormalities characteristic of many positive symptoms, which may have implications for treatment. For example, yoga, which promotes body awareness, has been shown to be effective in attenuating positive and negative symptoms and increasing social functioning in schizophrenia, exceeding the beneficial effects of aerobic exercise [42], [43].

### Conclusions

Patients with schizophrenia showed a stronger RHI, indexed by self-report and mislocalization of their own hand, suggesting that body ownership is disturbed in schizophrenia. Correlations between RHI strength and clinical symptoms and psychometric schizotypy suggest that disturbed body ownership is close to the heart of the schizophrenic experience. Moreover, our in-depth investigation of the spontaneous out-of-body experience induced by the RHI in R.M. provides rich descriptions of a context in which flexible and anomalous body ownership and representation could foster passivity delusions. These findings expand our etiological understanding of the most pathognomonic symptoms of schizophrenia and point towards innovative behavioral interventions.

## Supporting Information

Data S1 **Supplementary Data**. Methods, results, and discussion of a control experiment for temperature modulation during RHI induction.
(DOCX)
