# Supplementary material for: Disturbances in Body Ownership in Schizophrenia: Evidence from the Rubber Hand Illusion and Case Study of a Spontaneous Out-of-Body Experience
Source: PLoS One. 2011 Oct 31;6(10):e27089. doi: 10.1371/journal.pone.0027089 (PMC3205058; doi:10.1371/journal.pone.0027089)
Supplement: Data S1 — Supplementary Data. Methods, results, and discussion of a control experiment for temperature modulation during RHI induction. (DOCX) [file pone.0027089.s001.docx]

S1. Supplementary Data

To examine the possibility that skin temperature changes were due to specific conditions of our experimental set-up (e.g. different surfaces upon which subjects placed their stimulated and unstimulated hands) rather than to RHI induction, we performed a control experiment on a sample of 20 healthy undergraduate and graduate students (9 M / 11 F; age: 24.1 ± 4.4). The experimental apparatus and procedure was identical to the main study. However, no rubber hand was placed in the compartment adjacent to the real hand, and no stimulation was applied to the real hand. Temperature was measured at the same three points on both the hand inside the box, which would have been the stimulated hand, and the hand outside of the box, which would have been the unstimulated hand. These temperature measurements were taken at ‘baseline’ and 1,2, and 3 minutes following baseline. This procedure was repeated with the opposite hand inside the box; hand order was counterbalanced across subjects. Again, temperature was averaged across the three locations on both the hand inside and hand outside the box. The change in temperature for each hand was quantified as the difference in temperature between baseline and the average of the three post-baseline time points. A negative change indicated cooling relative to baseline.

A repeated measures ANOVA was performed on temperature change with position (inside or outside box) and target hand (hand placed inside the box) entered as factors. No main effect of target hand (F (1,19)=0.95, p=0.34) or target hand-by-position interaction effect (F (1,19)=1.45, p=0.24) was observed. There was a trend towards a main effect of position (F (1,19)=3.74, p=0.07), with a larger increase in temperature from baseline in the hand that was placed in the box (inside box: 0.52 ± 1.36°; outside box: 0.21 ± 1.33°). In contrast, during the main experiment the temperature of the stimulated hand, which was placed inside the box, *decreased* significantly; the results of this control study suggest that this pattern of cooling of the stimulated hand and warming of the unstimulated hand cannot be accounted for by the nature of the surface on which the two hands were placed.

Baseline temperatures in this control experiment were also examined. A repeated-measures ANOVA was performed on baseline temperature, with hand and position entered as factors. Replicating the finding from the main study, there was a significant main effect of hand (F (1,19)=12.52, p=0.002), with the left hand being warmer than the right hand. However, there was no significant main effect of position (F (1,19)=0.22, p=0.64) or hand-by-position interaction (F (1,19)=0.005, p=0.95). In the main study, the temperature of the stimulated hand, which was positioned inside the box, was warmer than the unstimulated hand at baseline. The lack of a significant position effect in this control study indicates that greater temperature in the stimulated hand at baseline might have been due to task-specific, rather than environment-specific, factors.

To summarize, the results of this control experiment indicate that the limb-specific temperature modulations observed in the main experiment were not caused by idiosyncratic parameters of the experimental environment, but instead, were related to RHI induction.
